# The Colombian Medical Oncologists Workforce

**DOI:** 10.1200/JGO.19.00221

**Published:** 2019-10-28

**Authors:** Raúl Murillo, Kelman Ojeda, Julio Solano, María Victoria Herrera, Oswaldo Sánchez

**Affiliations:** ^1^ Hospital Universitario San Ignacio, Bogotá, Colombia; ^2^ Pontificia Universidad Javeriana, Bogotá, Colombia

## INTRODUCTION

As a result of population aging, the absolute number of new cancer cases will increase in Colombia during the next decades from about 101,893 per year in 2018 (excluding nonmelanoma skin cancer) to about 136,246 per year in 2040, still with an average annual percentage change of −1.5%.^1^

The country shows an epidemiologic transition with breast and prostate cancers as leading causes of cancer incidence and mortality, and a mortality reduction from infection and tobacco-associated cancers such as those from the cervix, liver, stomach, larynx, esophagus, and lung.^2,3^ However, the latter remain highly prevalent and, with a few exceptions, population-based survival shows a relative reduction for most types of cancer and with significant differences when compared with high-income countries.^4^ Despite the reduced survival, the 5-year prevalence for all cancer types was 466.4 per 100,000 in 2018 corresponding to approximately 230,726 prevalent cases.^1^

The relative reduction in survival may indicate scarce progress in cancer early detection or proper timely treatment. In this regard, some analyses show a variable number of visits to the oncologist depending upon the stage of the disease, with higher rates at the beginning of treatment (particularly if neoadjuvant protocols are used), lower rates among survivors with controlled disease, and higher rates again toward the end of life.^5^ Thus, a high proportion of advanced cases at diagnosis, as may be the case in Colombia,^6^ would require greater oncologist time to meet the demand.

In addition to cancer incidence and stage at diagnosis, technologies used for cancer treatment also determine the demand of medical oncology. Currently target and immune therapies represent the highest investment in research and development by pharmaceutical companies,^7^ thus inducing permanent licensing of new oncology drugs and delivery of associated knowledge, which demands careful analysis by the medical oncologist workforce.

Moreover, new technologies lead to relevant changes in oncology practice; for instance, trastuzumab combined with cytotoxic drugs for the management of HER2-positive breast cancer patients (approximately 20% of cases) reduces relapse in 50% of cases and increases survival rates.^8^ Furthermore, the addition of trastuzumab increases treatment adverse effects and modifies the treatment schedule from approximately 8 to 27 sessions during the first year of treatment compared to chemotherapy alone.^5^ Similarly, systemic therapy combined with other treatment modalities, such as in the case of neoadjuvant and adjuvant protocols or concomitant chemo-radiation, the administration of several lines of treatment, and consolidation with bone marrow transplantation, have also shown better disease control and longer survival for different types of cancer, thus resulting in increased medical oncologist time for cancer care.

All factors described challenge the planning and supply of medical oncologist workforce, a situation critical to middle-income countries where better access to new cancer care technologies might take place, but the availability of resources to harmonize technology development with cancer care delivery is not a common situation. Hence, in this article, we review supply and demand of the medical oncologist workforce in Colombia using accredited sources of information and international standards.

## SUPPLY AND DEMAND OF MEDICAL ONCOLOGY

International standards regarding the medical oncologist workforce are variable; accordingly, while the Royal College of Physicians reports one full-time equivalent medical oncologist per 200 new cancer cases, New Zealand reports 1 per 210 cases (national standard 1 per 177 cases), and Australia one per 270 cases.^9^

In the international arena, a publication based on secondary sources found that 24% of 93 countries reported a rate of less than 150 new cancer cases per medical oncologist, 42% reported more than 500 cases, and 29% reported more than 1,000 cases, most of the latter were located in Africa where 7 countries reported not having any oncologist available. In Latin America rates of new cancer cases per oncologist fluctuate from 108 in Uruguay to 667 in Chile; however, all data from the region in the review derive from expert opinion, and consequently, their accuracy and reliability is low.^10^

In Colombia the National Observatory of Employment for Education reports 177 graduates in clinical oncology (solid tumors) up to 2016, and 153 graduates in hemato-oncology (solid tumors and hematologic malignancies), for a total of 324 specialists in the field of medical oncology (Table 1).^11^ Likewise, a verbal report by the Colombian Association of Hematology and Oncology (ACHO for its Spanish acronym), indicates approximately 180 clinical oncologists and 170 hematologists affiliated, the latter comprising hematologists (management of benign and malignant hematologic disease) and hemato-oncologists (management of benign and malignant hematologic diseases plus solid tumors). Yet, there is no available information on training abroad.

**TABLE 1:**
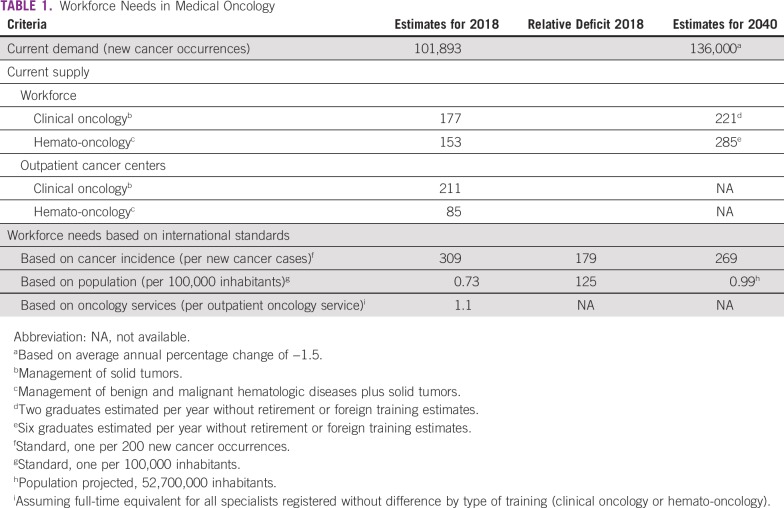
Workforce Needs in Medical Oncology

**CONTEXT**

**Key Objective**
Does the medical oncology workforce meet the current demand in Colombia?
**Knowledge Generated**
Colombia is a middle-income country with a growing economy. Available information suggest increased access to oncology care and oncology drugs; however, the medical oncology workforce does not follow the same pattern as represented in a relevant shortage according to international standards (deficit between 125 and 179 medical oncologists).
**Relevance**
Projections based on the number of new medical oncologists graduated per year in the country show a reduced shortage in the next 20 years but not enough to meet the demand. Better planning of the educational offer is highly desirable as well as collection of accurate data on characteristics of oncology practice in the country.

The data shows a workforce shortage deserving careful review regarding not only international standards but also the dynamic of oncology services in the country. According to the number of new cancer cases in 2018 (101,893 all cancer types and 9,360 hematologic malignancies),^1^ the rate of full-time equivalent specialists, counting both clinical oncologists and hemato-oncologists, would be 1 per 291 new cancer cases (Table 1). Accordingly, assuming unrealistically that all hemato-oncologists are dedicated full-time to oncology care, Colombia would need 20 additional specialists to meet a standard of 270 new cases per full-time equivalent, and 150 additional specialists to meet an ideal standard of 200 new cases per full-time equivalent. However, hematology practice involves the management of benign disease, which represents a substantially higher workload than cancer management; in addition, some clinical oncologists work with pharmaceutical companies and hold different administrative positions, all factors preventing exact estimates of time dedicated to clinical practice in cancer care.

From a different perspective, Australia reports 1.1 full-time equivalent medical oncologist per 100,000 inhabitants, the United Kingdom 1.3, and the United States 3.5.^12-14^ Furthermore, data from the United Kingdom indicate that 63.9% of the specialists rendered their services in more than 1 center, 19% were dedicated to a single type of cancer, 46% to maximum 2 types of cancer, 26% maximum to 3 types of cancer, and 8% were dedicated to more than 4 types of cancer.^13^ In the United States, the number of oncology centers has decreased while their size has increased and the number of visits per medical oncologist per week has decreased with some variation depending upon the type of practice (academic, private, or other) and age of the specialist (fewer visits in younger generations).^15^

In Colombia, by 2016, the National Registry of Health Service Providers (REPS for its Spanish acronym) reported 211 outpatient services in clinical oncology, 85 in hemato-oncology, and 175 in chemotherapy,^16^ indicating that the number of specialists in clinical oncology (solid tumors) is lower than the number of services officially registered for the medical specialty. The data suggest not only that medical oncologists render their services in several centers as in the United Kingdom, but also that some oncology services may not have a full-time specialist, and in fact, it might be possible that some services do not have any specialist available during several days of the week.

## SITUATION OF THE EDUCATIONAL OFFER

In the United States, according to the Electronic Residency Application Service (ERAS) of the Association of American Medical Colleges (AAMC), by 2019 there are about 145 training programs in hemato-oncology with a duration of 3 years, offered as a fellowship program after training in internal medicine.^17^ In Europe, the predominant modality of training in medical oncology is a basic medical specialty of 5 years duration and training in internal medicine as part of the academic program.^18^

In Latin America, most countries have at least one training program in medical oncology management of both solid tumors and hematologic malignancies. In Colombia, according to the National Higher Education Information System (SNIES), there is currently 1 training program in clinical oncology, 3 programs in hemato-oncology, and 2 programs in hematology.^11^ All of them require preliminary training of 3 years in internal medicine, and their duration is 2 years for training in clinical oncology and hematology, and 3 years for training in hemato-oncology.

With 5 to 10 graduates per year from the existing programs in clinical oncology and hemato-oncology, it would take between 15 and 30 years to meet the national demand, without counting retirements and without considering the expected increase in the absolute number of new cancer cases over the period.

## DISCUSSION

Colombia, with 45.5 million inhabitants, would require 450 specialists to obtain 1 full-time equivalent medical oncologist per 100,000 inhabitants, which represents a shortage of approximately 100 medical oncologists (Table 1). Furthermore, according to the National Registry of Health Service Providers (REPS), five Colombian districts do not have any type of oncology service.^16^

By 2012, there were 748 health care centers with oncology services available in the country,^19^ a figure that drops down to 443 in 2017 with an increase in the average number of oncology services per center (ie medical oncology, radiotherapy, oncology surgery, palliative care, etc.).^20^ Similarly, in the United States a reduction in the number but increase in size of oncology centers has been observed^15^; however, in Colombia the number of new cases per year by oncology center remains low (between 55 in La Guajira and 309 in Casanare).^20^

In the United States reasons related to lifestyle and greater complexity in the management of cancer patients, with the requirement of a more comprehensive approach to the disease, have been linked to the dynamic of oncology services observed.^15^ In Colombia, the number of clinical oncology services (solid tumors) is greater than the number of specialists in this field, thus suggesting a complex dynamic with either low expertise, given the low number of cancer cases per center, or coverage with partial times, and even absence of a specialist during several days of the week in some centers.

Regarding the educational offer, in the United States there is 1 training program in hemato-oncology per 2.2 million inhabitants, whereas in Colombia there is 1 per 11.2 million inhabitants (1 per 7.5 if considering training in hematology). Despite differences in cancer incidence (age-standardized rates for all cancer types 352.2 and 178.8 for the United States and Colombia, respectively)^1^ and the higher rate of full-time equivalent specialist in the United States compared with other high-income countries, the educational offer seems low in Colombia. Given the different training modalities (clinical oncology, hemato-oncology, hematology), it is not possible to make a definite conclusion on the medical oncologist workforce; however, data from official registries (ACHO and SNIES) show a workforce shortage that does not meet international standards, and is having a negative influence on the quality of cancer care. Therefore, the gathering of detailed data through a structured survey among ACHO affiliates would be an asset for planning more effective training in the field in Colombia.
